# Redetermination of the crystal structure of RhPb_2_ from single-crystal X-ray diffraction data, revealing a rhodium deficiency

**DOI:** 10.1107/S2056989021012275

**Published:** 2021-11-25

**Authors:** Takashi Mochiku, Yoshitaka Matsushita, Nikola Subotić, Takanari Kashiwagi, Kazuo Kadowaki

**Affiliations:** a National Institute for Materials Science, 1-2-1 Sengen, Tsukuba, Ibaraki 305-0047, Japan; b University of Tsukuba, 1-1-1 Tennouda, Tsukuba, Ibaraki 305-8573, Japan

**Keywords:** crystal structure, rhodium, lead, inter­metallic compound, deficiency, superconductivity

## Abstract

RhPb_2_ (rhodium dilead) is a superconductor crystallizing in the CuAl_2_ structure type (space group *I*4/*mcm*). The Rh and Pb atoms are located at the 4*a* (site symmetry 422) and 8*h* (*m*.2*m*) sites, respectively. The crystal structure is composed of [RhPb_8_] anti­prisms, which share their square faces along the *c* axis and the edges in the direction perpendicular to the *c* axis. We have succeeded in growing single crystals of RhPb_2_ and have re-determined the crystal structure on basis of single-crystal X-ray diffraction data. In comparison with the previous structure studies using powder X-ray diffraction data [Wallbaum (1943 ▸). *Z. Metallkd*. **35**, 218–221; Havinga *et al.* (1972 ▸). *J. Less-Common Met.* **27**, 169–186], the current structure analysis of RhPb_2_ leads to more precise unit-cell parameters and fractional coordinates, together with anisotropic displacement parameters for the two atoms. In addition and likewise different from the previous studies, we have found a slight deficiency of Rh in RhPb_2_, leading to a refined formula of Rh_0.950 (9)_Pb_2_.

## Abstract 2

The unit-cell parameters and inter­atomic distances of RhPb_2_ are different from those of the previous studies using powder X-ray diffraction, and Rh is found to be deficient.

## Chemical context

A large number of binary inter­metallic compounds with the CuAl_2_ structure type have been reported (Wallbaum, 1943 ▸; Havinga *et al.*, 1972 ▸; Havinga, 1972 ▸), and several of them exhibit superconductivity (Gendron & Jones, 1962 ▸). RhPb_2_ is one of them, with a superconducting transition temperature (*T*_c_) of 2.66 K (Gendron & Jones, 1962 ▸). *β*-RhPb_2_ adopting the *β*-PdBi_2_ structure type (space group *I*4/*mmm*) has been reported as a candidate material for topological superconductors (Zhang *et al.*, 2019 ▸), and RhPb_2_ crystallizing in the CuAl_2_ structure type has also attracted much attention. While the previous powder X-ray studies of RhPb_2_ (Wallbaum, 1943 ▸; Havinga *et al.*, 1972 ▸) used polycrystalline material prepared by a melting method, we have grown RhPb_2_ single crystals by application of a vertical pulling mechanism using an infrared mirror furnace. Here we report on the redetermined crystal structure of RhPb_2_ based on single-crystal X-ray data.

## Structural commentary

**Figure 1:**
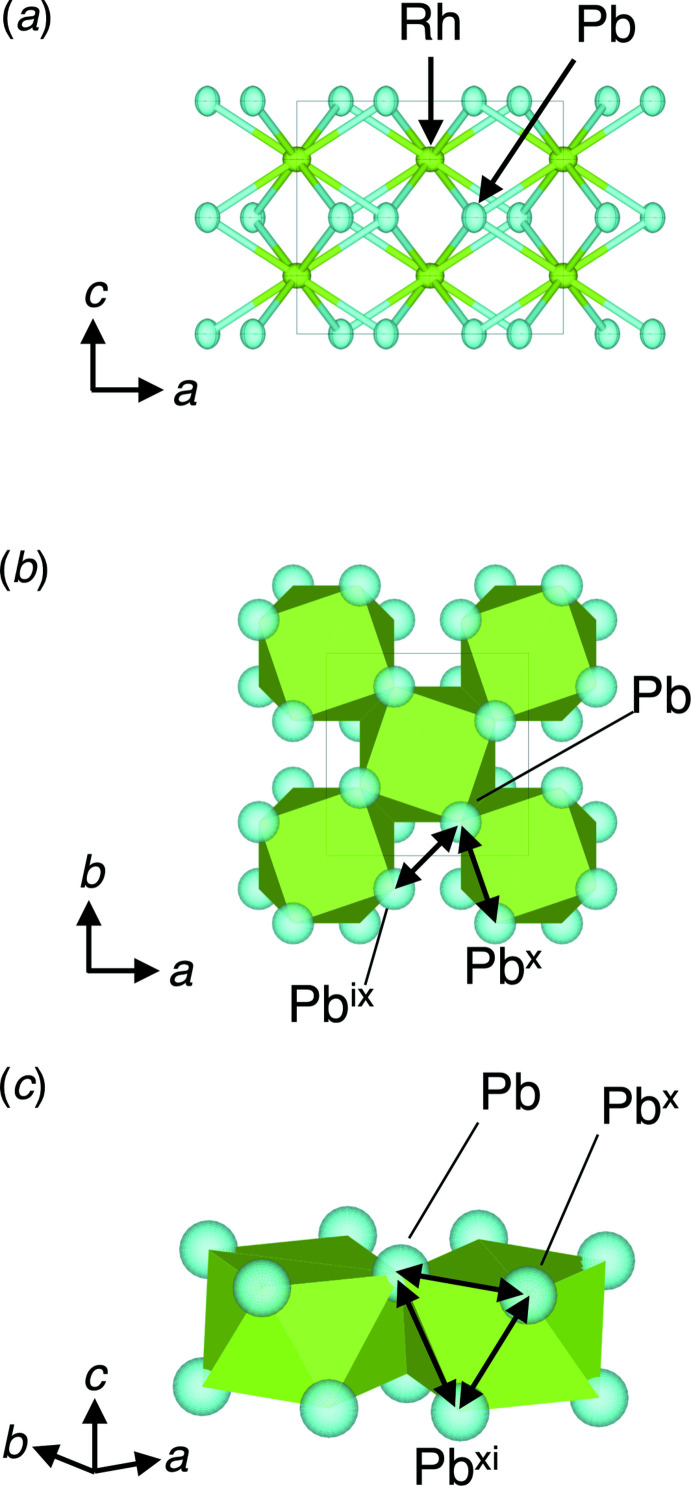
(*a*) The crystal structure of RhPb_2_ in a view along the *b* axis showing the atoms in the asymmetric unit with displacement ellipsoids at the 99.9%; (*b*) the crystal structure of RhPb_2_ in polyhedral representation in a view along the *c* axis; (*c*) details of the linkage of the [RhPb_8_] anti­prisms in the crystal structure of RhPb_2_.

The crystal structure of RhPb_2_ refined from single-crystal data is essentially the same as determined previously (Wallbaum, 1943 ▸; Havinga *et al.*, 1972 ▸). RhPb_2_ is composed of [RhPb_8_] anti­prisms, which share the square faces along the *c* axis and the edges in the direction perpendicular to the *c* axis (Fig. 1 ▸). The Rh atom (site symmetry 422) is surrounded by eight Pb atoms occupying the edges of the [RhPb_8_] anti­prism, and two Rh atoms are spaced along the *c* axis at a distance of half of the unit-cell parameter *c*. The Pb—Pb distance in the adjacent [RhPb_8_] anti­prism is the shortest among the Pb—Pb distances (Table 1 ▸; Fig. 1 ▸ *b*, Pb—Pb^ix^); all Pb—Pb distances belonging to the shared square faces of the [RhPb_8_] anti­prism are equal (Fig. 1 ▸ *b*, Pb—Pb^x^), while the Pb—Pb distances belonging to the sides of the triangle of the [RhPb_8_] anti­prism are all different (Fig. 1 ▸ *c*, Pb—Pb^x^, Pb—Pb^xi^ and Pb^x-^–Pb^xi^).

While RhPb_2_ has been reported to be single phase only in a Pb-deficient sample (Havinga *et al.*, 1972 ▸), we have found a deficiency of Rh rather than a deficiency of Pb in the grown single crystals. The chemical composition obtained from the analysis of the occupancy of Rh is Rh_0.950 (9)_Pb_2_. Hamilton’s *R*-factor ratio test (Hamilton, 1965 ▸) was used to compare the *R* factors for the models with and without a deficiency of Rh. The result rejected the model without deficiency of Rh at a significance level of less than 0.005.

Table 1 ▸ shows the unit-cell parameters and inter­atomic distances obtained from the current and the previous studies (Wallbaum, 1943 ▸; Havinga *et al.*, 1972 ▸). The unit-cell parameters are more precise and larger than those of the previous studies, and the free fractional coordinate of Rh was also obtained with higher precision. The resulting inter­atomic distances are slightly different from those in the previous studies. Anisotropic displacement parameters, which were not reported previously, were also obtained from the current redetermination.

## Synthesis and crystallization

Single crystals of RhPb_2_ were grown from the Pb-rich melt (molar ratio Rh:Pb = 1:8) by a slow cooling process in a steep temperature gradient infrared furnace according to the Pb–Rh binary phase diagram (El-Boragy *et al.*, 1992 ▸), where RhPb_2_ is shown to grow through the peritectic reaction incongruently melting between 593 and 913 K. The raw materials of Rh and Pb were of 99.9% purity in powder form (300 mesh) and 99.99% in shots, respectively, purchased from Furuuchi Chemical Co. Prior to crystal growth, Rh and Pb were melted together in an evacuated silica tube by heating with a flame torch. The obtained ingot was then put into a new silica tube and was vacuum sealed. The silica tube was hung in an infrared mirror furnace, which generally has a strong temperature gradient around the focal point. The sample silica tube was heated above 913 K, where the sample became completely liquid. Then, the silica tube was placed at the position where the temperature gradient is the highest. The silica tube was rotated slowly (∼10 r.p.m.) to promote single crystals to grow in a uniform temperature horizontally with a steep temperature gradient vertically. A silica tube with a cone-shaped bottom was used. The furnace temperature was slowly decreased with a constant rate of 0.5 K h^−1^ until it reached the temperature well below 593 K, then it was lowered to room temperature.

**Figure 2:**
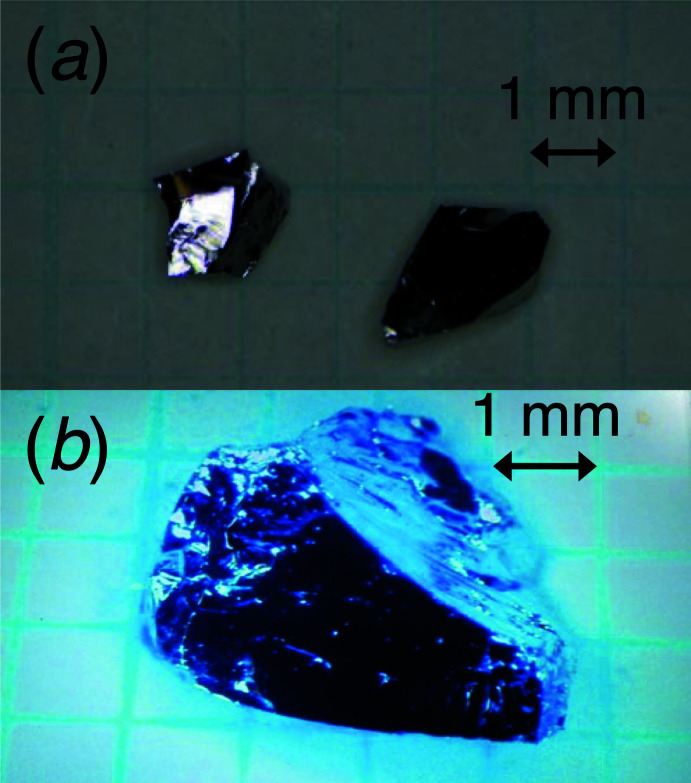
(*a*) A photograph of two pieces of single crystals of RhPb_2_ taken under an optical microscope; (*b*) an enlarged optical photograph of one of the single-crystals of RhPb_2_.

After removing the silica tube carefully, the grown boule showed clearly the liquid–solid phase boundary as a horizontal line in the upper part of the boule, indicating that the single-crystal growth had progressed as planned according to the phase diagram (El-Boragy *et al.*, 1992 ▸). More than half of the grown boule from the bottom appeared to have turned into a single crystal of RhPb_2_. The latter cleaves easily, showing shiny reflection with a silvery luster from the cleaved surface. The single crystal was rather soft and could easily be scratched by tweezers. The product seems to be stable in air because the color of the cleaved surface did not change over time. In Fig. 2 ▸ photographs of the grown single crystals of RhPb_2_ are shown.

## Refinement

Crystal data, data collection and structure refinement details are summarized in Table 2 ▸. The equivalent isotropic atomic displacement parameter (*U*_eq_) of the Rh sites for the model without deficiency of Rh was 0.0131 (5) Å^2^, which was larger than that of Pb (0.0107 (3) Å^2^). We refined the occupancies of Rh and Pb. While the refined occupancy of Pb was very close to full occupation, the refined occupancy of Rh indicated a significant deficiency of this site. The final *wR*(*F* ^2^) value for the model without deficiency of Rh was 0.047, and that for the model with deficiency of Rh was 0.042. In the final model [occupancy of Rh = 0.950 (9); full occupancy of Pb] the atomic displacement parameter (*U*_eq_) of the Rh site is the same as that of the Pb site.

## Supplementary Material

Crystal structure: contains datablock(s) I, global. DOI: 10.1107/S2056989021012275/wm5626sup1.cif

Structure factors: contains datablock(s) I. DOI: 10.1107/S2056989021012275/wm5626Isup4.hkl

CCDC reference: 2123239

Additional supporting information:  crystallographic information; 3D view; checkCIF report

## Figures and Tables

**Table 1:** Comparison of unit-cell parameters and inter­atomic distances (Å) at room temperature in RhPb_2_ determined in previous and the present studies

	Wallbaum (1943 ▸)	Havinga *et al.* (1972 ▸)	This work
*a*	6.651 (3)	6.674 (3)	6.7068 (4)
*c*	5.853 (3)	5.831 (3)	5.8623 (6)
Rh—Pb	2.902	2.885 (6)	2.9016 (2)
Pb—Pb^ix^	2.972	3.134 (14)	3.1313 (13)
Pb—Pb^*x*^	3.544	3.520 (10)	3.5416 (4)
Pb—Pb^xi^	3.603	3.662 (9)	3.6734 (6)
Pb^*x*^—Pb^xi^	3.400	3.319 (7)	3.3448 (7)

Symmetry codes: (ix) −*x* + 1, −*y*, −*z* + 1; (*x*) −*y* + 1, *x* − 1, *z*; (xi) *y* + {1\over 2}, −*x* + {1\over 2}, −*z* + {1\over 2}.

**Table 2:** Experimental details

Crystal data	
Chemical formula	Rh_0.95_Pb_2_
*M*_r_	512.14
Crystal system, space group	Tetragonal, *I*4/*m* *c* *m*
Temperature (K)	295
*a*, *c* (Å)	6.7068 (4), 5.8623 (6)
*V* (Å^3^)	263.69 (4)
*Z*	4
Radiation type	Mo *K*α
μ (mm^−1^)	132.87
Crystal size (mm)	0.11 × 0.05 × 0.03

Data collection	
Diffractometer	XtaLAB Mini II
Absorption correction	Multi-scan (*CrysAlis PRO*; Rigaku OD, 2019 ▸)
*T*_min_, *T*_max_	0.123, 1.000
No. of measured, independent and observed [*I* > 2σ(*I*)] reflections	461, 117, 88
*R*_int_	0.033
(sin θ/λ)_max_ (Å^−1^)	0.708

Refinement	
*R*[*F* ^2^ > 2σ(*F* ^2^)], *wR*(*F* ^2^), *S*	0.021, 0.042, 1.01
No. of reflections	117
No. of parameters	9
Δρ_max_, Δρ_min_ (e Å^−3^)	1.96, −1.56

Computer programs: *CrysAlis PRO* (Rigaku OD, 2019 ▸), *SHELXT* (Sheldrick, 2015*a* ▸), *SHELXL* (Sheldrick, 2015*b* ▸) and *OLEX2* (Dolomanov *et al.*, 2009 ▸).

## supplementary crystallographic information

### Crystal data

**Table:**

Rh_0.95_Pb_2_	*D*_x_ = 12.900 Mg m^−^^3^
*M**_r_* = 512.14	Mo *K*α radiation, λ = 0.71073 Å
Tetragonal, *I*4/*m**c**m*	Cell parameters from 295 reflections
*a* = 6.7068 (4) Å	θ = 6.1–30.2°
*c* = 5.8623 (6) Å	µ = 132.87 mm^−^^1^
*V* = 263.69 (4) Å^3^	*T* = 295 K
*Z* = 4	Irregular, metallic dark grey
*F*(000) = 827	0.11 × 0.05 × 0.03 mm

### Data collection

**Table:**

XtaLAB Mini II diffractometer	88 reflections with *I* > 2σ(*I*)
Detector resolution: 10.0000 pixels mm^-1^	*R*_int_ = 0.033
ω scans	θ_max_ = 30.2°, θ_min_ = 4.3°
Absorption correction: multi-scan (CrysAlisPro; Rigaku OD, 2019)	*h* = −9→8
*T*_min_ = 0.123, *T*_max_ = 1.000	*k* = −7→9
461 measured reflections	*l* = −8→7
117 independent reflections	

### Refinement

**Table:**

Refinement on *F*^2^	Primary atom site location: iterative
Least-squares matrix: full	*w* = 1/[σ^2^(*F*_o_^2^) + (0.013*P*)^2^] where *P* = (*F*_o_^2^ + 2*F*_c_^2^)/3
*R*[*F*^2^ > 2σ(*F*^2^)] = 0.021	(Δ/σ)_max_ < 0.001
*wR*(*F*^2^) = 0.042	Δρ_max_ = 1.96 e Å^−^^3^
*S* = 1.01	Δρ_min_ = −1.56 e Å^−^^3^
117 reflections	Extinction correction: SHELXL2018/3 (Sheldrick 2015b), Fc^*^=kFc[1+0.001xFc^2^λ^3^/sin(2θ)]^-1/4^
9 parameters	Extinction coefficient: 0.0030 (3)
0 restraints	

### Special details

**Table:**

Geometry. All esds (except the esd in the dihedral angle between two l.s. planes) are estimated using the full covariance matrix. The cell esds are taken into account individually in the estimation of esds in distances, angles and torsion angles; correlations between esds in cell parameters are only used when they are defined by crystal symmetry. An approximate (isotropic) treatment of cell esds is used for estimating esds involving l.s. planes.

### Fractional atomic coordinates and isotropic or equivalent isotropic displacement parameters (Å^2^)

**Table:**

	*x*	*y*	*z*	*U*_iso_*/*U*_eq_	Occ. (<1)
Rh	0.500000	0.500000	0.750000	0.0103 (7)	0.950 (9)
Pb	0.66507 (7)	0.16507 (7)	0.500000	0.0103 (3)	

### Atomic displacement parameters (Å^2^)

**Table:**

	*U*^11^	*U*^22^	*U*^33^	*U*^12^	*U*^13^	*U*^23^
Rh	0.0111 (8)	0.0111 (8)	0.0088 (11)	0.000	0.000	0.000
Pb	0.0091 (3)	0.0091 (3)	0.0128 (4)	0.0013 (3)	0.000	0.000

### Geometric parameters (Å, º)

**Table:**

Rh—Rh^i^	2.9312 (3)	Rh—Pb^viii^	2.9016 (2)
Rh—Rh^ii^	2.9312 (3)	Pb—Pb^ix^	3.1313 (13)
Rh—Pb^iii^	2.9016 (2)	Pb—Pb^x^	3.5416 (4)
Rh—Pb^iv^	2.9016 (2)	Pb—Pb^xi^	3.6734 (6)
Rh—Pb^ii^	2.9016 (2)	Pb—Pb^xii^	3.5416 (4)
Rh—Pb	2.9016 (2)	Pb—Pb^iv^	3.5416 (4)
Rh—Pb^v^	2.9016 (2)	Pb—Pb^vii^	3.5416 (4)
Rh—Pb^vi^	2.9016 (2)	Pb—Pb^xiii^	3.3448 (7)
Rh—Pb^vii^	2.9016 (2)	Pb—Pb^v^	3.3448 (7)
			
Rh^ii^—Rh—Rh^i^	180.0	Rh^v^—Pb—Pb^xi^	99.966 (13)
Pb^ii^—Rh—Rh^ii^	59.663 (4)	Rh^xiv^—Pb—Pb^x^	52.390 (2)
Pb—Rh—Rh^i^	120.337 (4)	Rh^ii^—Pb—Pb^xii^	96.44 (2)
Pb^iii^—Rh—Rh^i^	59.663 (4)	Rh^xiv^—Pb—Pb^iv^	148.841 (9)
Pb^iv^—Rh—Rh^i^	120.337 (4)	Rh^v^—Pb—Pb^xii^	52.390 (2)
Pb^vi^—Rh—Rh^i^	59.663 (4)	Rh^xiv^—Pb—Pb^xi^	50.729 (10)
Pb^viii^—Rh—Rh^i^	59.663 (4)	Rh^v^—Pb—Pb^vii^	96.44 (2)
Pb^vii^—Rh—Rh^ii^	59.663 (4)	Rh—Pb—Pb^xii^	96.44 (2)
Pb^vi^—Rh—Rh^ii^	120.337 (4)	Rh—Pb—Pb^x^	148.842 (9)
Pb^iii^—Rh—Rh^ii^	120.337 (4)	Rh^v^—Pb—Pb^xiii^	107.993 (18)
Pb—Rh—Rh^ii^	59.663 (4)	Rh^ii^—Pb—Pb^ix^	106.118 (12)
Pb^vii^—Rh—Rh^i^	120.337 (4)	Rh—Pb—Pb^ix^	106.118 (12)
Pb^v^—Rh—Rh^ii^	120.337 (4)	Rh^xiv^—Pb—Pb^vii^	96.44 (2)
Pb^v^—Rh—Rh^i^	59.663 (4)	Rh^v^—Pb—Pb^ix^	106.118 (12)
Pb^viii^—Rh—Rh^ii^	120.337 (4)	Rh^ii^—Pb—Pb^x^	148.841 (9)
Pb^iv^—Rh—Rh^ii^	59.663 (4)	Rh^xiv^—Pb—Pb^ix^	106.118 (12)
Pb^ii^—Rh—Rh^i^	120.337 (4)	Rh^ii^—Pb—Pb^xi^	93.646 (8)
Pb^vii^—Rh—Pb^iii^	135.14 (2)	Rh^v^—Pb—Pb^v^	54.805 (6)
Pb^iv^—Rh—Pb^vii^	119.326 (8)	Rh—Pb—Pb^iv^	52.390 (2)
Pb^iv^—Rh—Pb^viii^	70.390 (11)	Rh^xiv^—Pb—Pb^v^	107.993 (18)
Pb^ii^—Rh—Pb^iii^	70.390 (11)	Rh—Pb—Pb^vii^	52.390 (2)
Pb^iv^—Rh—Pb^ii^	75.220 (4)	Rh—Pb—Pb^xiii^	107.993 (18)
Pb^iv^—Rh—Pb^v^	135.14 (2)	Rh^ii^—Pb—Pb^v^	107.993 (18)
Pb—Rh—Pb^viii^	78.54 (2)	Rh^ii^—Pb—Pb^xiii^	54.805 (6)
Pb^vii^—Rh—Pb^ii^	75.220 (4)	Rh^ii^—Pb—Pb^vii^	52.390 (2)
Pb^vi^—Rh—Pb	135.14 (2)	Rh^ii^—Pb—Pb^iv^	52.390 (2)
Pb^iv^—Rh—Pb^iii^	78.54 (2)	Rh^xiv^—Pb—Pb^xiii^	54.805 (6)
Pb^vi^—Rh—Pb^iii^	75.220 (4)	Pb^xiii^—Pb—Pb^vii^	64.40 (2)
Pb^viii^—Rh—Pb^ii^	135.14 (2)	Pb^v^—Pb—Pb^xii^	64.40 (2)
Pb—Rh—Pb^iii^	147.76 (2)	Pb^xii^—Pb—Pb^xi^	101.180 (5)
Pb^vi^—Rh—Pb^viii^	119.326 (8)	Pb^x^—Pb—Pb^iv^	127.53 (3)
Pb^viii^—Rh—Pb^iii^	75.220 (3)	Pb^ix^—Pb—Pb^xi^	64.773 (7)
Pb^iv^—Rh—Pb	75.220 (3)	Pb^vii^—Pb—Pb^xi^	124.800 (14)
Pb^iv^—Rh—Pb^vi^	147.76 (2)	Pb^xii^—Pb—Pb^iv^	142.47 (3)
Pb—Rh—Pb^vii^	75.220 (3)	Pb^iv^—Pb—Pb^xi^	101.180 (5)
Pb—Rh—Pb^ii^	119.326 (8)	Pb^vii^—Pb—Pb^xii^	52.47 (3)
Pb^viii^—Rh—Pb^vii^	147.76 (2)	Pb^ix^—Pb—Pb^vii^	153.764 (14)
Pb^v^—Rh—Pb^viii^	75.220 (4)	Pb^xiii^—Pb—Pb^xi^	60.398 (9)
Pb^vi^—Rh—Pb^ii^	78.54 (2)	Pb^ix^—Pb—Pb^x^	63.764 (14)
Pb—Rh—Pb^v^	70.390 (12)	Pb^v^—Pb—Pb^vii^	64.40 (2)
Pb^vi^—Rh—Pb^vii^	70.390 (11)	Pb^vii^—Pb—Pb^iv^	90.0
Pb^v^—Rh—Pb^ii^	147.76 (2)	Pb^ix^—Pb—Pb^v^	118.80 (2)
Pb^v^—Rh—Pb^iii^	119.326 (8)	Pb^v^—Pb—Pb^x^	102.295 (2)
Pb^v^—Rh—Pb^vii^	78.54 (2)	Pb^ix^—Pb—Pb^xiii^	118.80 (2)
Pb^vi^—Rh—Pb^v^	75.220 (3)	Pb^xiii^—Pb—Pb^xii^	64.40 (2)
Rh—Pb—Rh^v^	109.610 (11)	Pb^v^—Pb—Pb^xiii^	122.41 (4)
Rh^v^—Pb—Rh^ii^	147.76 (2)	Pb^xiii^—Pb—Pb^x^	102.295 (2)
Rh—Pb—Rh^ii^	60.674 (7)	Pb^ix^—Pb—Pb^xii^	153.764 (14)
Rh^ii^—Pb—Rh^xiv^	109.610 (12)	Pb^v^—Pb—Pb^xi^	154.764 (7)
Rh—Pb—Rh^xiv^	147.76 (2)	Pb^ix^—Pb—Pb^iv^	63.764 (14)
Rh^v^—Pb—Rh^xiv^	60.674 (8)	Pb^vii^—Pb—Pb^x^	142.47 (3)
Rh^v^—Pb—Pb^x^	52.390 (2)	Pb^v^—Pb—Pb^iv^	102.295 (2)
Rh—Pb—Pb^xi^	150.417 (3)	Pb^x^—Pb—Pb^xi^	55.200 (14)
Rh—Pb—Pb^v^	54.805 (6)	Pb^xiii^—Pb—Pb^iv^	102.295 (2)
Rh^v^—Pb—Pb^iv^	148.841 (9)	Pb^xii^—Pb—Pb^x^	90.0
Rh^xiv^—Pb—Pb^xii^	52.390 (2)		

Symmetry codes: (i) −*x*+1, −*y*+1, −*z*+2; (ii) −*x*+1, −*y*+1, −*z*+1; (iii) *x*−1/2, *y*+1/2, *z*+1/2; (iv) *y*, −*x*+1, −*z*+1; (v) −*x*+3/2, −*y*+1/2, −*z*+3/2; (vi) *y*+1/2, −*x*+3/2, −*z*+3/2; (vii) −*y*+1, *x*, *z*; (viii) −*y*+1/2, *x*−1/2, *z*+1/2; (ix) −*x*+1, −*y*, −*z*+1; (x) −*y*+1, *x*−1, *z*; (xi) *y*+1/2, −*x*+1/2, −*z*+1/2; (xii) *y*+1, −*x*+1, −*z*+1; (xiii) −*x*+3/2, −*y*+1/2, −*z*+1/2; (xiv) *x*+1/2, *y*−1/2, *z*−1/2.
